# An Ultra-Fast TSP on a CNT Heating Layer for Unsteady Temperature and Heat Flux Measurements in Subsonic Flows

**DOI:** 10.3390/s22020657

**Published:** 2022-01-15

**Authors:** Martin Bitter, Michael Hilfer, Tobias Schubert, Christian Klein, Reinhard Niehuis

**Affiliations:** 1 Institute of Jet Propulsion, Bundeswehr University Munich, Werner-Heisenberg-Weg 39, 85577 Neubiberg, Germany; tobias.schubert@unibw.de (T.S.); reinhard.niehuis@unibw.de (R.N.); 2 Institute of Aerodynamics and Flow Technology, German Aerospace Center, Bunsenstraße 10, 37073 Göttingen, Germany; michael.hilfer@dlr.de (M.H.); christian.klein@dlr.de (C.K.)

**Keywords:** ultra-fast temperature-sensitive paint, time-resolved heat transfer, subsonic cylinder flow, vortex shedding modes

## Abstract

In this paper, the authors demonstrate the application of a modified Ru(phen)-based temperature-sensitive paint which was originally developed for the evaluation of unsteady aero-thermodynamic phenomena in high Mach number but short duration experiments. In the present work, the modified TSP with a temperature sensitivity of up to −5.6%/K was applied in a low Mach number long-duration test case in a low-pressure environment. For the demonstration of the paint’s performance, a flat plate with a mounted cylinder was set up in the High-Speed Cascade Wind Tunnel (HGK). The test case was designed to generate vortex shedding frequencies up to 4300 Hz which were sampled using a high-speed camera at 40 kHz frame rate to resolve unsteady surface temperature fields for potential heat-transfer estimations. The experiments were carried out at reduced ambient pressure of $p_{\infty }$ = 13.8 kPa for three inflow Mach numbers being $Ma_{\infty }=\left[0.3;0.5;0.7\right]$. In order to enable the resolution of very low temperature fluctuations down to the noise floor of $10^{-5}$ K with high spatial and temporal resolution, the flat plate model was equipped with a sprayable carbon nanotube (CNT) heating layer. This constellation, together with the thermal sensors incorporated in the model, allowed for the calculation of a quasi-heat-transfer coefficient from the surface temperature fields. Besides the results of the experiments, the paper highlights the properties of the modified TSP as well as the methodology.

## 1. Introduction

The non-intrusive detection of surface temperature and pressure fields was boosted in the last decades through the introduction and further development of coatings containing sensitive luminophores. A wide variety of temperature- and pressure-sensitive paints is currently available, covering nearly all fields of experimental fluid mechanics ranging from microscopic to macroscopic and from low-speed to high Mach number flows as recently summarized in Liu et al. [1]. Nearly 40 years ago, the development started with paints which were able to measure steady or quasi-steady distributions of surface quantities. Through the last years, the rising demands on unsteady data for validating modern time-resolved computational methods such as Large-Eddy Simulation has motivated the development of new paints capable of providing this data. Matsumura et al. are one of the pioneers in measuring quantitative unsteady temperature fields [2]. A review of recent achievements in the field of unsteady PSP & TSP is given in [3].

Unfortunately, the majority of available luminophores used for these paints has a certain sensitivity to both, pressure and temperature, which somehow affects the accuracy of the final result [1]. To reduce pressure dependency in a TSP, the luminophores are usually encapsulated into binder materials such as poly-amide (PA), poly-urethan (PU) or nylon (Ny), resulting in higher layer thicknesses which reduce the response time of the active layer due to increased temperature penetration time through the binder. In contrast, these compositions allow for stronger emission intensities and better signal-to-noise ratio (SNR).

The original version of the ultra-fast-response TSP used in the present work, was initially designed for its application in short-duration facilities generating high enthalpy flows such as the High Enthalpy Shock Tunnel Göttingen (HEG) [4,5,6,7]. Typically, such facilities allow for short-duration flow experiments in the order of milliseconds, but expose strong temperature gradients. These applications call for TSPs which can react ultra fast but do not necessarily need to be very sensitive because the strong temperature gradients provide high SNR.

The original TSP had a temperature sensitivity of about −3%/K, being a slightly more sensitive kind of paint compared to other luminophores such as Europium or Ruthenium complexes. Ozawa and Lawrence [8] employed Ru(phen) as luminophor embedded in an ethanol-soluble PA-based polymer to measure the short-duration heat flux on a generic re-entry capsule during supersonic flow in a shock tube. They resolved flow structures in the low microsecond range as the sampling rates were as high as 180 kHz during their tests. Such high recording rates determine low integration times which, in turn, require enormous amounts of optical excitation power. As examined for organic silicon polymers by Sharma et al. [9], a strong increase in excitation energy typically is closely linked to the raise of introduced thermal energy which both cause a remarkable photo-degradation of the luminophores even during the short duration experiment.

Apart from high-enthalpy flow facilities, TSPs are widely used in large industrial wind tunnels, e.g., for transition detection of boundary layers on airfoils or wings. A heated model surface is widely used to increase the SNR in such a TSP application. Klein et al. [10,11], developed a sprayable heating layer based on carbon nanotubes (CNT) which can be applied in a homogeneous and even layer onto a model surface. A voltage is applied to the CNT layer, whose high electrical resistance leads to a well controllable and homogeneously heated surface.

The main intention of this paper is to introduce a modified version of the original TSP, which is well suited for long-lasting experiments in subsonic flows. The goal is to combine an increased paint sensitivity with a CNT heating layer to reach high SNR. This paper will demonstrate, (a) the ability of that TSP to resolve high-frequency but low-temperature fluctuations under subsonic flow conditions and, (b) that these (unsteady) surface temperature fields are suited for quantitative heat transfer estimations. The following sections introduce paint specific characteristics as well as the experimental demonstration test case, which is a cylinder mounted on a flat plate and being exposed to cross flow.

## 2. Modified Fast-Response Temperature Sensitive Paint

As stated above, for the desired test cases in this paper, both, the sensitivity and the SNR of the TSP had to be improved to enable the resolution of low-temperature fluctuations. In [12], Schramm and Hilfer reported on the experimental investigation of response time of similar Ru(phen) and polyamide-based type TSP with temperature sensitivities above −5%/K and overall TSP-Layer response time τ around 3 μs. The utilized Ru(phen) was the Dichlorotris (1,10-phenanthroline) Ruthenium(II) hydrate 98%, with a lifetime of 1 μs.

**Figure 1:**
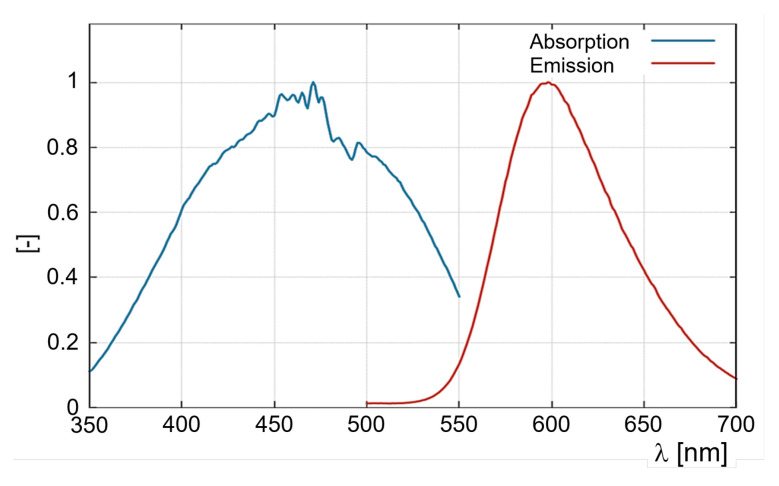
Emission and excitation spectra of Ru(phen) embedded in the nylon matrix which was dissolved in ethanol to be spayed on a sample by spray gun.

Typical absorption and emission spectra of the luminophor are shown in Figure 1. Ru(phen) can be excited over a range of wavelengths with a maximum around 450–460 nm. Emission maximum of Ru(phen) is found around 600 nm. Based on the investigations in [12] an implementation of this TSP-system was considered for subsonic and transonic facilities with highly unsteady flows. Until recently, most of the TSP-investigations in sub- and transonic wind tunnels concentrated on steady flows using luminophores with relatively long response time of around 100 μs in case of Europium-based luminophores, compare listed values in the appendix of Liu et al. [1]. Additionally, the luminophores are usually embedded in relatively thick active layers above 30 μm which allows for higher emission intensities but reduces the response time of the active layer due to high temperature penetration time through the active layer. Recently, Miozzi et al. [13] showed the ability of Europium based paint to resolve unsteady flow phenomena in a water channel with frequencies up to 200 Hz. Natural wall temperature variance between different boundary flow regimes is usually quite low and can be calculated according to [14] using the recovery factor *r* for turbulent and laminar flow as follows:

(1) $$T_{aw}=T_{\infty }\cdot [1+\frac{r(\gamma -1)}{2}\cdot Ma_{\infty }^{2}]$$

where $r_{tur}\propto Pr^{1/3}$ is the turbulent and $r_{lam}\propto Pr^{1/2}$ the laminar recovery factor. In order to increase the temperature variance, a temperature difference between the model and the flow has to be applied so the convective heat transfer coefficient α can be employed instead of recovery factor calculation with $\alpha_{tur}>\alpha_{lam}$[15]. This can be accomplished by heating or cooling the model or the medium. In this work, a heating of the model surface is accomplished using spray-able carbon nanotubes (CNT) heating layer as developed by Klein et al. [10,11]. The thickness of the CNT layer being ≈40 μm was adjusted according to a desired electrical resistance. Applying a voltage, which typically ranges up to 120 V, heats up the surface. The conducting CNT layer must be insulated from the top surface against accidental contact by experimenters and against short circuit from metal parts inside the model. Therefore, the CNT-Layer was embedded in between two uniformly distributed PU-based insulation layers of about 20 μm thickness each.

**Figure 2:**
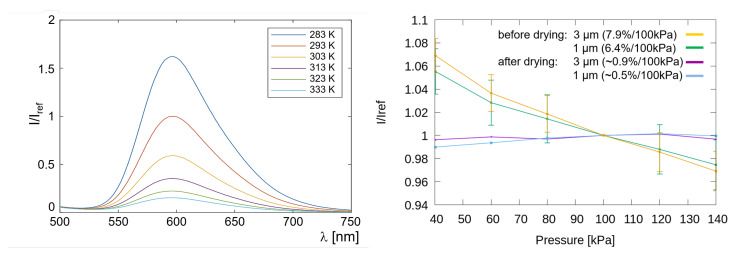
(**Left**) Emission spectra for TSP calibrated at $p_{ref}$ = 100 kPa for various temperatures. (**Right**) pressure dependency of TSP for different layer thicknesses, before and after drying and illuminating, from [17].

In order to resolve fast flow changes using TSP three main characteristics have to be evaluated and optimized. For one, the thickness of the active layer has to be reduced to a minimum. In the present setup, the thickness of the active layer, which was applied by a conventional Sata HVLP spray gun with 1 mm nozzle diameter, is measured to be around 1 μm ± 0.2 μm using a Perthometer (Mahr Perthometer S2 with MarSurf GD 25). The surface roughness of the active layer was measured to be $R_{a}\approx$ 0.1 μm. With thinner layers the emission intensities are significantly reduced assuming constant excitation intensity. For the emission to be still detectable, better cameras or more powerful excitation sources are required. The second consideration is the temperature sensitivity of the active layer, where higher temperature sensitivity allows for a resolution of lower temperature changes. Also, flow characteristics which are faster than the complete temperature diffusion time of the TSP layer can be resolved, since even a partial diffusion is still detectable in this case. In a series of tests, Schmid et al. [16] found the optimal paint composition for highest temperature sensitivity to be at −5.4%/K. The emission spectra changing with calibration temperature are depicted on the left in Figure 2 for the most promising composition.

Last but not least, an important consideration is the pressure dependency of the luminophor. Ru(phen)-based luminophores are usually pressure sensitive. For a luminophor to be pressure sensitive it has to have the ability to transfer energy from the exited molecule to the oxygen molecule without generating an emission photon in the visible range. In the present case the luminophores are embedded in a nylon matrix with very low oxygen permeability. The polymer was modified by the vendor to be soluble in ethanol. On the right in Figure 2 the pressure dependency of the modified paint is presented. Freshly applied to a paint sample and immediately calibrated in a static calibration chamber, a 3 micron active layer shows a maximal pressure dependency of up to ≈8%/100 kPa. A second set of samples was allowed to dry for several hours under dark ambient conditions. After drying and restrained illumination, the pressure dependency of the sample with 1 micron active layer dropped to about 0.5%/100 kPa. The next chapter presents the paint characteristics which were examined during the in-situ calibration directly on the model in the test section.

## 3. Test Environment and Methodology

### 3.1. Experimental Setup

**Figure 3:**
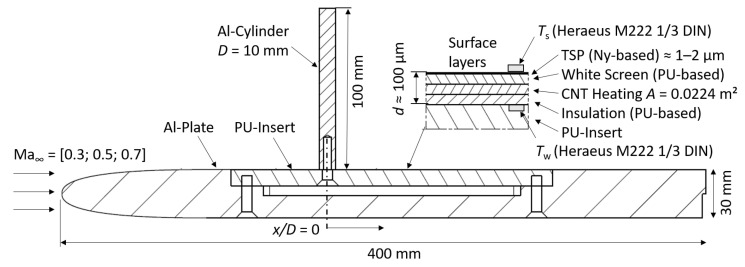
Flat-plate test specimen for the high-speed TSP measurements in the wind tunnel. Layer dimensions not to scale.

For the demonstration of the paint’s ability to resolve high-frequency low-temperature fluctuations in subsonic flows, a flat plate wind tunnel model made from aluminum was used, as illustrated in Figure 3. The measurements were carried out on the surface of a poly-urethane (PU) part, inserted in the upper surface of the model. The PU-insert has a machined pocket of 100 μm depth which holds the various coating layers as sketched in the detail of the figure. Two thin copper film conductors were integrated into the pocket to evenly supply the electrical heating power to the carbon nanotube layer, see [11].

Two static RTDs of type Heraeus M222 1/3 DIN with 0.1 K accuracy were used to determine reference temperatures during the TSP measurements. One sensor ($T_{s}$) was fixed on top of the measurement surface using arctic silver glue. The installation was located outside the interesting wake flow field because it formed a tiny bump of about 1 mm height which may also slightly affect the accuracy of the measured recovery temperature. The second sensor ($T_{w}$) was integrated in the PU-insert, just underneath the insulation layer. From practical reasons, both sensors were located at a distance of x/D=7 downstream of the cylinder. An aluminum cylinder of *D* = 10 mm is mounted approx. 135 mm downstream of the leading edge. It has a height of 100 mm which widely impeded strong 3d-interaction with the tip. The cylinder was electrically insulated against the first surface layer to prevent a short circuit with the high currents from the heating layer. However, this thin electrical insulation does not provide a high thermal insulation, which can lead to thermal currents from or into the cylinder as discussed further in the next section. The cylinder is supposed to generate high-frequency temperature fluctuations, which will be captured in the ensuing TSP measurements.

The experiments in this work were conducted in the High-Speed Cascade Wind Tunnel (HGK) of the Institute of Jet Propulsion at the Bundeswehr University Munich [18]. The HGK test facility is a continuously operating, open loop wind tunnel. The main components of the wind tunnel are located inside a cylindrical pressure chamber, which enables an independent Mach and Reynolds number variation through the variation of the ambient pressure down to approx. 3.5 kPa at minimum.

**Figure 4:**
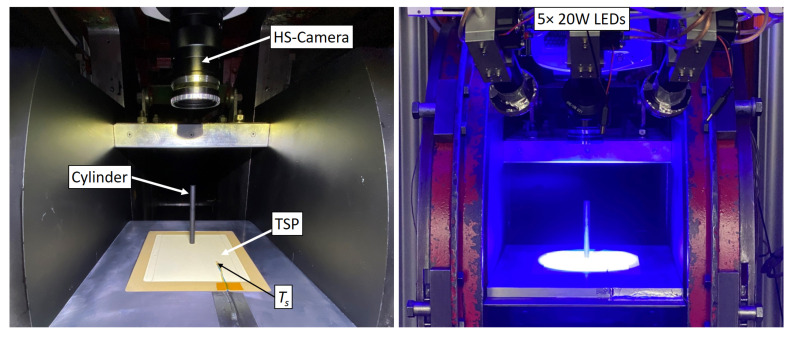
Experimental setup and test equipment for the high-speed TSP measurements in the wind tunnel facility.

The flat plate, as it was set up in the wind tunnel test section including the required test equipment, is shown in Figure 4. Five high-power LED emitters (type Luminus CBT-120) with 20 W optical output each were equipped with band pass filters with $\lambda_{LED}$ = 450 ± 40 nm and focused on the region of interest for a homogeneous TSP excitation even at low integration time. The high-speed camera (type Phantom V2640) for image data acquisition was equipped with a 570 nm long-pass filter. It was set up at a working distance of about 300 mm from the flat plate and was tuned to capture the TSP fluorescence at a frame rate of 40 kHz over a sequence length of 1 s.

### 3.2. Methodology

#### 3.2.1. Operating Conditions

As one of the main intentions of the test series was to demonstrate the ability of the TSP to precisely resolve low-amplitude surface temperature fluctuations in order to enable time-resolved heat transfer measurements in sub sonic flows, the tests were conducted for three different inflow Mach numbers $Ma_{\infty }=\left[0.3;0.5;0.7\right]$ at a constant ambient pressure of 13.8 kPa. The changing inflow conditions relate to different Reynolds numbers based on the diameter of the cylinder. All aerodynamic operating conditions are listed in Table 1. The Prandtl number was assumed constant at Pr = 0.74.

#### 3.2.2. TSP Calibration

The intensity response which was finally applied for the calculation of the temperature field was in-situ calibrated directly on the flat plate model. In order to re-check for a potential pressure-dependency of the paint as outlined above, the calibration was performed at atmospheric pressure $p_{\infty }$ = 95.3 kPa and at operating pressure $p_{\infty }$ = 13.8 kPa while the wind tunnel was at rest.

**Figure 5:**
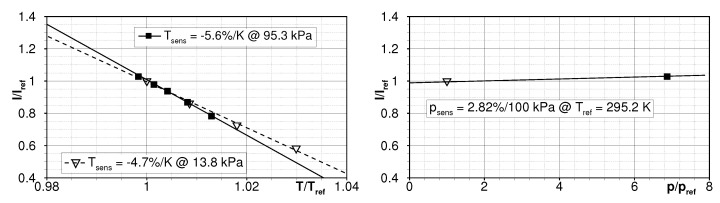
In-situ calibration curves for Ru(phen)-based Temperature Sensitive Paint under different ambient pressures showing the temperature sensitivity (**left**) and the pressure dependency (**right**).

Figure 5 shows the in-situ calibration curves, where the surface temperature was tuned by heating the model and the intensity data was picked in the vicinity of the surface temperature sensor $T_{s}$ which served as reference. The reference condition was at ambient pressure $p_{ref}$ = 13.8 kPa and surface temperature $T_{ref}=22\;{\;}^{\circ }$C. The Ru(phen)-based TSP applied to the model exhibits its highest sensitivity of about −5.6%/K at atmospheric conditions. However, even under low-pressure conditions it is remarkably high at about −4.7%/K. Contrary to the results presented in the section above, the applied paint composition still exhibits a slightly higher pressure dependency of 2.8%/100 kPa after drying and before testing. It is expected that the drying time was slightly less compared to the paint samples due to the tight test schedule. Due to the fact that the experiments were carried out under low pressure conditions and under the assumption that the pressure fluctuations in the cylinder wake are 15% of the dynamic pressure according to [19], this would lead to maximum pressure amplitudes of about 750 Pa, compare Table 1. At the available sensor saturation of about 2300 cts, the pressure fluctuations would lead to intensity fluctuations below 0.5 cts (i.e., 0.48 cts) which cannot be adequately resolved by the camera. Accordingly, the pressure dependency is expected to have no relevance on the accuracy of the TSP temperature fields from these demonstration tests.

#### 3.2.3. Image Acquisition and Data Reduction

The TSP suffers degradation from intense excitation resulting in reduced luminosity, which was the main motivation to keep the acquisition sequence short (i.e., in the range of seconds) and thereby enable testing at multiple operating points. The 40 kHz camera frame rate (i.e., 40,000 wind-on images over 1 s sequence length; 1000 images at reference) determined an effective sensor area of 333×1530 px${}^{2}$. The integration time $t_{i}$ was varied between 24μs for $Ma_{\infty }=0.3$ and 15μs for $Ma_{\infty }=0.7$. The LEDs were allowed to warm up for 0.1 s prior of the image acquisition and operated continuously during the sequence. All relevant image system conditions are listed in Table 2.

Each operating point was started with a wind-on image set at $q_{el}^{\prime \prime }=0$ followed by up to 4 image sets of various heating settings. Directly after the last heating point was measured, the TSP reference images were acquired under wind-off conditions. During each set point, all relevant parameter—including the start/stop trigger of the imaging system and the LED trigger were logged in the wind tunnel data system. This sensor data was up-sampled and made available at every single intensity image through post-processing. The minimum and maximum heating settings are listed in Table 3.

The original spatial resolution of the intensity images was reduced by a factor of 16 (i.e., 4 pixel in *x*- & *y*-direction were binned together) in order to handle the whole data stack in memory for image processing. An effect of the binning on fundamental findings in the results is not expected because all dominant structures in the flow were still resolved as shown later in the Mode 1 energy spectra.

The absolute surface temperature $T_{TSP}$ was retrieved from a second order polynomial in-situ fit which was applied to the intensity ratio $I_{ref}/I$ at each set point. The offset between the raw TSP temperatures and the $T_{s}$ readings was calculated in the vicinity of $T_{s}$ which was still visible in the field of view (FOV). This offset was simply subtracted from the entire raw TSP temperature map.

Every test case was mainly analyzed in the frequency domain in order to emphasize the paint’s ability to measure high-frequency but low-amplitude temperature fluctuations. To recover the local amplitudes, a standard windowed FFT as implemented in Matlab was used. The full signal (40,000 samples) was chopped into consecutive segments of 4000 samples. The segments had an overlap of 50% (i.e., 2000 samples). Hence, 19 segments were Fourier-transformed and finally averaged. The frequency resolution was 10 Hz. Additionally, the usage of the spectral proper orthogonal decomposition (SPOD), as presented by Towne [20], enables the separation of dominant characteristic modes in the flow field and calculates their spatial and temporal development. This method was applied to reconstruct the dominant vortex shedding modes expected to occur at a reduced frequency (or Strouhal number) of Sr≈0.2. The spatial and temporal resolution of these dominant shedding frequencies and even their higher harmonics called for the high-kilohertz frame rates.

#### 3.2.4. Heat Transfer Coefficient

It is known, that precise heat transfer coefficient (HTC) measurements using heated surfaces are challenging with respect to the precise distinction of the conductive, convective and radiative portions of the heat flux, compare e.g., [21,22,23]. The work of Estorf [24] emphasizes the calculation of the complex multi-lateral heat fluxes through a finite model in order to estimate high-accuracy heat fluxes from image-based temperature fields. The individual portions sum up to the total electric power introduced into the system as follows:

(2) $$q_{el}^{\prime \prime }=q_{conv}^{\prime \prime }+q_{cond}^{\prime \prime }+q_{rad}^{\prime \prime }.$$

The quantitative heat transfer coefficient α is expressed by:

(3) $$\alpha =\frac{q_{conv}^{\prime \prime }}{T_{TSP}-T_{aw}}$$

by means of the convective exchange between the surface and the flow $q_{conv}^{\prime \prime }$, the measured surface temperature $T_{TSP}$ and the adiabatic wall temperature $T_{aw}$. Anyhow, since the methodology behind a fully comprehensive estimation of all portions is rather complex and would be out of the scope of the work presented here, the simple and widely used Fourier law will be applied. The authors are aware that there are multiple approaches which can be used in order to improve the accuracy of the measured HTC. Unfortunately, plenty of them are computationally highly demanding, such as proposed e.g., by [21]. The Fourier law as used in Temperature-Sensitive Paint applications e.g., by [2,22,25,26] for estimating $q_{conv}^{\prime \prime }$ formulates as follows:

(4) $$q_{conv}^{\prime \prime }=k/d\cdot \left(T_{w}-T_{TSP}\right)$$

where *k* is the thermal conductivity, *d* the thickness of the corresponding heated layer and $T_{w}$ the internal wall temperature. In order to keep the conductive losses into the model small and, in turn, assure validity of a 1 d heat transfer model at a first glance, low thermal conductivity of the model’s base material is essential. The chosen PU (Obomodulan Sahara) for the insert exhibits a high mechanical strength to withstand the aerodynamic forces and it has a reasonable low thermal conductivity of *k* = 0.233 W/mK compared to aluminum whose value is about thousand times larger. Hence, it is expected, that the electrical heating power nearly completely turns into convective heat flux. The conductive and radiative fluxes $q_{cond}^{\prime \prime }$ and $q_{rad}^{\prime \prime }$ were neglected.

The adiabatic wall temperature is usually retrieved from a local surface pressure or Mach number distribution correlated with the boundary layer condition by using the according recovery factor in Equation (1). As the flat plate model suffered from missing surface pressure taps and as the investigated flow field is widely three-dimensional and highly dynamic, this approach was not productive here. Hence, the steady $T_{aw}$ distribution was estimated from the averaged temperature fields for various heating settings at each aerodynamic operating point as follows:

1. An electric voltage setting was applied to the CNT layer which results in a heated surface. The model was allowed to stabilize its conditions which were monitored by the internal and external sensors $T_{w}$ and $T_{s}$.
2. The heat flux $q_{el}^{\prime \prime }$ was calculated from the current and voltage given by the power supply according to $q_{el}^{\prime \prime }=U\cdot I/A_{CNT}$.
3. The surface temperature was measured with TSP and averaged at each $q_{el}^{\prime \prime }$ set point.

**Figure 6:**
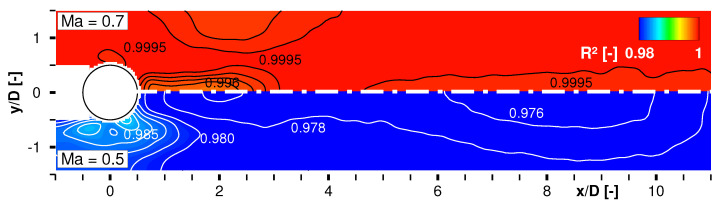
Determination coefficient $R^{2}$ of linear fit $T_{TSP}$-vs.-$q_{el}^{\prime \prime }$ for estimation of the adiabatic wall temperature $T_{aw}$ compared for $Ma_{\infty }=0.7$ and $Ma_{\infty }=0.5$.

Finally, a linear fit function $T_{TSP}$-vs.-$q_{el}^{\prime \prime }$ was applied to every pixel in the field of view. The intersect value of each fit at $q_{el}^{\prime \prime }=0$ delivered the local adiabatic wall temperature. Figure 6 presents the determination coefficient $R^{2}$ as a measure of the goodness of the linear fit for $Ma_{\infty }=0.5$ and $Ma_{\infty }=0.7$. (Note: for the $Ma_{\infty }=0.3$ test case the authors performed two other heating settings apart from the adiabatic case $q_{el}^{\prime \prime }=0$. Hence, the determination coefficient for a linear approximation is $R^{2}=1$ per definition.)

The plot shows a very good correlation $R^{2}\to 1$ between the heating power and the measured surface temperature, slightly better for $Ma_{\infty }=0.7$. As a consequence of the cylinder being made from aluminum and the weak thermal insulation, a slightly different fit quality and hence a larger uncertainty in the estimated adiabatic wall temperature is present in the near vicinity of the cylinder. The white area upstream of the cylinder were masked out due to shadowing.

## 4. Results

**Figure 7:**
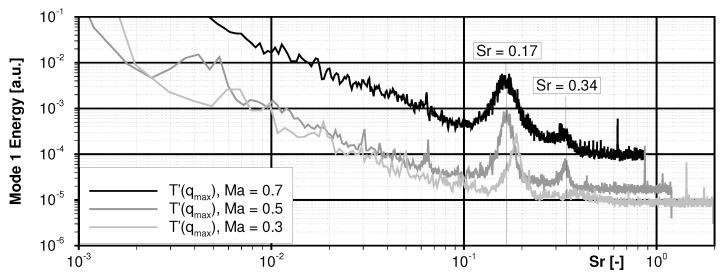
Mode 1 energy spectra for all inflow Mach numbers calculated by means of a spectral proper orthogonal decomposition.

At first, a result of the SPOD analysis shows the $T^{\prime }$ amplitude spectrum of the mode with the highest energy content in Figure 7 for all three test Mach numbers. All spectra reveal a dominant peak in the region of Sr≈0.17 and a second weaker one around Sr≈0.34. The Strouhal numbers were calculated based one the shedding frequency $f_{shed}$, the cylinder diameter *D* and the free-stream velocity $u_{\infty }$ from Table 1. Most likely, the representative velocity might not be the inflow velocity in this case but slightly less. Based on the fact, that TSP measures a wall-bounded footprint of flow structures which widely move inside the boundary layer of the turbulent cylinder wake, the Sr values may be slightly less than the expected values of 0.2, compare [19,27]. The sudden expansion of the open test section downstream of the pitot probe at the test section inlet may be another reason. Anyhow, for this Ru(phen)-based TSP it can be stated that the noise floor for detecting temperature amplitudes is in the order of $10^{-5}$ K when using averaging spectral approaches. (Note: For $Ma_{\infty }=0.3$, the peak at Sr=0.34 highly likely collapses with the noise floor and cannot be clearly resolved.)

**Figure 8:**
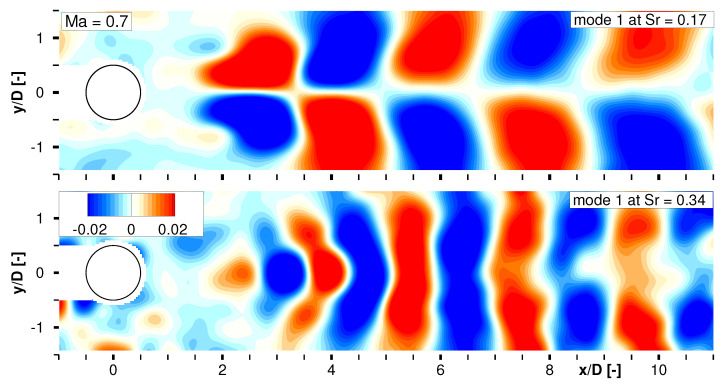
Reconstruction of characteristic flow patterns at Sr=0.17 (**top**) and Sr=0.34 (**bottom**) for $Ma_{\infty }=0.7$.

As a second result of the SPOD analysis, the spatial development of the dominant vortex shedding and its higher harmonic was calculated for $Ma_{\infty }=0.7$ as exemplary depicted in Figure 8. The pattern in the upper plot shows a highly resolved alternating von-Kármán vortex street in the wake, whereas the dominant structures start to establish from x/D≈2. Their inclined appearance is the consequence of the local convection velocity which is lower inside and higher outside of the cylinder wake. The lower plot displays the reconstructed footprint from the first harmonic of the coherent structures as they develop at Sr=0.34. Halving of the structures’ spatial dimension that coincides with a doubled frequency is clearly observable comparing both plots. Reconstructed time series data showing the temporal development of the characteristic modes can be provided as supplementary material with this paper.

**Figure 9:**
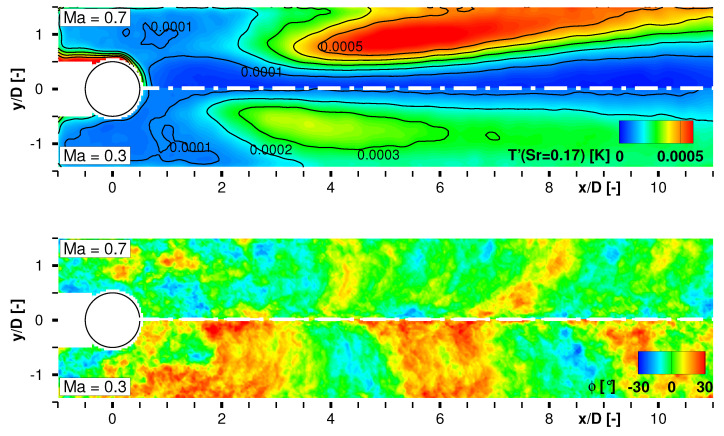
Local temperature fluctuation amplitudes (**top**) and phase angle ϕ (**bottom**) at Sr=0.17 compared for $Ma_{\infty }=0.3$ and $Ma_{\infty }=0.7$.

The local temperature fluctuation amplitudes in the FOV at Sr=0.17 for the highest and lowest test Mach number close the spectral analysis of the coherent structures as well as the discussion of the paint’s potential to resolve low-amplitude temperature fluctuations. Figure 9 shows the fluctuation amplitudes and the corresponding phase angles as a result of the windowed FFT comparing $Ma_{\infty }=0.3$ and $Ma_{\infty }=0.7$. Both, higher fluctuation amplitudes and a downward shifted position of the local maximum is expected and evident for $Ma_{\infty }=0.7$. The widening of the wake which is coupled with an outward transportation of the dominant structures was clearly resolved. The evaluation potential of the available data set is fairly inexhaustible. Unfortunately, a deeper fluid-mechanical interpretation would be out of scope of the present paper but will be focused in a projected paper following this work.

**Figure 10:**
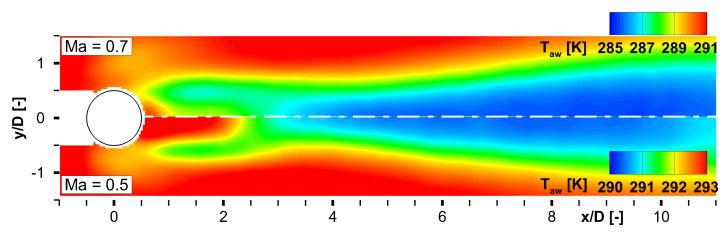
Adiabatic wall temperature distribution in the wake of the cylinder compared for $Ma_{\infty }=0.7$ and $Ma_{\infty }=0.5$.

Finally, the measured surface heat transfer will be discussed, whose estimation from the TSP fields was the second major goal of this campaign. The discussion is started with the adiabatic wall temperature fields, which were calculated from the local fit method outlined above. Figure 10 shows the result of the $T_{TSP}$-vs.-$q_{el}^{\prime \prime }$ fitting for $Ma_{\infty }=0.5$ and $Ma_{\infty }=0.7$. The field plots for $T_{aw}$ show a strong foot print of the 3-d wake flow. Note the individual color levels of both plot halfs.

**Figure 11:**
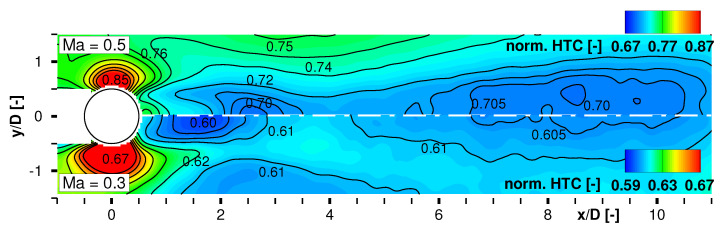
Quasi heat transfer coefficient $\mathrm{norm}.\;\mathrm{HTC}=\left(T_{w}-T_{TSP}\right)/\left(T_{TSP}-T_{aw}\right)$ compared for $Ma_{\infty }=0.5$ and $Ma_{\infty }=0.3$ at maximum el. heat flux $q_{el,max}^{\prime \prime }$.

The temperatures in the outer parts of the FOV at x/D≈4 are in good agreement with the theoretically calculated adiabatic wall temperature at the surface sensor for which the corresponding inflow Mach number and a recovery factor of r=0.91 (turbulent boundary layer) was relevant. The wake flow regime exhibits a lower adiabatic wall temperature compared to the undisturbed flow field due to the strong turbulent mixing between the outer flow at a static temperature of $T_{\infty }=\left[279;266\right]$ K, respectively for $Ma_{\infty }=\left[0.5;0.7\right]$, and the thermal boundary layer. The temperature field develops widely symmetric whereas the core of the cylinder wake extends up to x/D≈2.5. Figure 11 shows the comparison of the local thermal fluxes in the cylinder wake between $Ma_{\infty }=0.3$ and $Ma_{\infty }=0.5$ at the maximum heating set point $q_{el,max}^{\prime \prime }$. The displayed quantity $\left(T_{w}-T_{TSP}\right)/\left(T_{TSP}-T_{aw}\right)$ can be interpreted as normalized or quasi-HTC without acknowledging the material’s thermal conductivity and the accurate wall thickness. Note that the upper half of the plot has a different color level compared to the lower part. The overall contour level is higher for $Ma_{\infty }=0.5$, as expected. As the aspired layer thickness *d* is very thin but could only be estimated since PU being the non-conductive carrying material, small deviations in the thickness estimation would have an enormous effect on the quantitative HTC value. Knowing *d* and *k* precisely would end up in a scaling of the shown thermal flux distributions, but, unfortunately is a major topic for accurate heat transfer measurements as outlined above.

The thermal fluxes are highest in the region of the strongest flow acceleration at the outboard sides of the cylinder for both Mach numbers. Vice versa, it is lowest in the central wake region. Further downstream from x/D>4, the thermal flux values remain widely constant.

**Figure 12:**
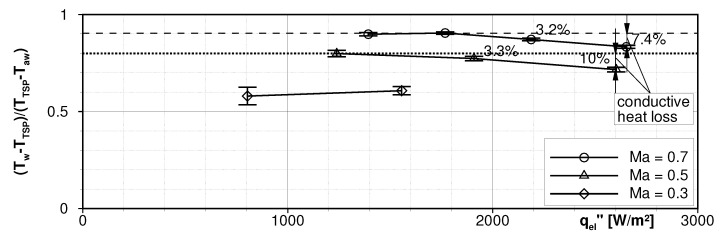
Integral quasi-HTC for all tested operating points including conductive loss approximation.

Figure 12 collects the integral normalized HTC values for all operating points. For this plot, the HTC map as displayed in the previous figure was calculated for each operating point and averaged over the entire FOV. The integral values were plotted versus the electrical heat flux $q_{el}^{\prime \prime }$. The expected trend of rising HTC with increasing Mach number is clearly evident. In case the assumption of the simple Fourier law holds, the HTC should be constant per definition throughout each Mach number for every electrical heat flux as indicated by the dotted line for $Ma_{\infty }=0.5$ or the dashed line for $Ma_{\infty }=0.7$, respectively. As the HTC values decrease slightly for raised heating powers, it is concluded, that the internal conductive loss flux $q_{cond}^{\prime \prime }$ cannot be neglected. The heat loss was estimated in a first glance from the percentage drop of the HTC values as indicated by the numbers in the figure. Calculating the slopes for the losses at both Mach numbers, conductive losses of about 9.5% per 1 kW/m${}^{2}$ heating power were estimated.

As stated above, this paper is entirely focused on the measurement of high-frequency but low-temperature fluctuation and the usage of such fields to recover heat transfer coefficients. The motivation of this paper does not raise a claim in a low-uncertainty heat flux estimation, at all. The former two were demonstrated with convincing results as discussed so far. In case the latter is of interest, there were some approaches mentioned above but many more can be found in literature. In principle, the application of the proposed method, so far, enables the calculation of unsteady HTC-fields as well. Anyhow, the fundamental discussion of such results and their comparison to the steady HTC fields would not deliver any deeper substantial insights. Therefore, it will be avoided at this point. In any way it should be noted that the test specimen, which will be used for such measurements and which was equipped with steady RTDs during the experiments presented here, should be equipped with reference thermocouples being able to follow the high-frequency temperature fluctuations - at least on the model surface. This would allow for the precise determination of high-frequency surface temperatures or HTC, similar as presented for unsteady PSP by Bitter et al. [28].

## 5. Summary and Outlook

This paper presents the characteristics and performance of a modified version of an ultra-fast Ru(phen)-based temperature-sensitive paint whose composition was tuned to resolve low-temperature but high-frequency flow events in subsonic flows. The modified paint revealed a higher temperature sensitivity of up to −5.6%/K and a fairly low pressure dependency of 2.8%/100 kPa.

An experimental test series was carried out at subsonic inflow conditions and reduced ambient pressure of 13.8 kPa in the High-Speed Cascade Wind Tunnel of the Bundeswehr University in Munich. For the demonstration of the paint’s potential, a cylinder of *D* = 10 mm was mounted on a flat plate model and exposed to inflow Mach numbers ranging from $Ma_{\infty }=0.3$–0.7. The test case was designed to generate vortex shedding frequencies up to 4300 Hz which had to be recognized by the modified TSP. The wall-bounded temperature footprint around the cylinder and in the wake was measured with a high-speed TSP system capturing intensity images at 40,000 Hz frame rate.

The generation of a sufficiently high SNR, which enables the detection of the low-temperature fluctuations during the TSP measurement, was realized applying a sprayable carbon nanotube heating layer underneath the active coating. The CNT layer which acts like an electrical resistance and consequently heats up if a voltage is applied. Doing so, a heating power up to 2600 W/m${}^{2}$ was applied to the CNT layer to increase the temperature difference between the flow and the model surface by up to 10 K.

The results clearly reveal that the utilized combination from the modified ultra-fast TSP with the CNT-layer was generating sufficient SNR which enables the detection of temperature fluctuations down to a noise floor of $10^{-5}$ K. The frame rate of 40 kHz was sufficiently high to resolve and reconstruct the pattern of dominant coherent structures over all investigated subsonic flow Mach numbers.

Special attention was devoted to the derivation of the (unsteady) heat transfer coefficient from the measured TSP fields. The authors were aware of the challenge and potential methods to calculate high precision heat fluxes. However, a first-order Fourier approach was applied at a first glance to generate a quasi-HTC from the measured temperature fields and a temperature sensor incorporated into the model. Especially, the integral quasi-HTC values revealed that there were conductive losses of around 9.5% per 1 kW/m${}^{2}$ heat flux which cannot be neglected as presumed in the 1d Fourier approach. The consideration of these conductive losses by applying a sophisticated but more demanding approach may improve the HTC results significantly in future test campaigns.

This paper impressively presents the benefits of the Ru(phen)-based TSP which has high sensitivity and enables the detection of low temperature fluctuations in subsonic flows in combination with a CNT heating layer. It was also shown, that this is an attractive method to measure unsteady HTCs. At all, the discussion of a quantitative HTC with enhanced measurement accuracy was accepted to be far off the scope of this paper. Same holds for a deeper fluid-mechanical interpretation of the cylinder flow and its characteristics. This is projected to happen in an upcoming paper. 

## Figures and Tables

**Table 1:** Aerodynamic operating conditions for the high-speed TSP measurements. Adiabatic wall temperatures assume turbulent boundary layer states at the position of the reference sensor and a recovery factor of r=0.91 for all operating points.

${\mathrm{Ma}}_{\infty }$	ReD	$p_{\infty }$ [kPa]	$q_{\infty }$ [kPa]	$p_{t}$ [kPa]	$u_{\infty }$ [m/s]	$T_{t}$ [K]	$T_{\mathrm{aw}}$ [K]	$f_{\mathrm{shed}}$ [Hz]
0.3	95k	13.8	0.89	14.7	102.1	293.15	292.7	1850
0.5	165k	13.8	2.57	16.4	167.4	293.15	291.9	2760
0.7	245k	13.8	5.35	19.1	229.5	293.15	290.8	4320

**Table 2:** Operating conditions of the imaging system for the high-speed TSP measurements.

FOV [px]	Frame Rate [Hz]	$t_{\mathrm{seq}}$ [s]	$\lambda_{\mathrm{LED}}$ [nm]	$\lambda_{\mathrm{em}}$ [nm]	$t_{i}$ [μs]
333×1530	40,000	1	450±40	570 LP	[15;20;24]

**Table 3:** Min./max. thermal conditions for the individual inflow conditions.

${\mathrm{Ma}}_{\infty }$	*U* [V]	*I* [A]	$q_{\mathrm{el}}^{\prime \prime }$ [W/m${}^{2}$]	$T_{s}$ [K]	$T_{w}$ [K]
0.3	$\left[0;85\right]$	$\left[0;0.41\right]$	$\left[0;1556\right]$	$\left[294.8;307.5\right]$	$\left[295.4;317.1\right]$
0.5	$\left[0;110\right]$	$\left[0;0.53\right]$	$\left[0;2603\right]$	$\left[293.3;308.2\right]$	$\left[293.7;321\right]$
0.7	$\left[0;110\right]$	$\left[0;0.54\right]$	$\left[0;2652\right]$	$\left[290.6;302.1\right]$	$\left[291.1;313\right]$

## Data Availability

Selected data can be provided upon request.

## Abbreviations

The following abbreviations are used in this manuscript:

CNT	Carbon nanotubes
FFT	Fast Fourier transformation
FOV	Field of view
HGK	High-Speed Cascade Wind Tunnel of the Bundeswehr University Munich
HTC	Heat transfer coefficient, quasi-HTC = $\frac{T_{w}-T_{TSP}}{T_{TSP}-T_{aw}}$
LED	Light-emitting diode
Ny	Nylon
PA, PU	Poly-amide, poly-urethane
P-/TSP	Pressure-/Temperature-Sensitive Paint
Ru(phen)	Dichlorotris (1,10-phenanthroline) Ruthenium(II) hydrate 98%
RTD	Resistant temperature detector
SNR	Signal-to-noise ratio
SPOD	Spectral proper orthogonal decomposition (after [20])

## Symbols

α	Heat transfer coefficient $=k/d\cdot \frac{T_{w}-T_{TSP}}{T_{TSP}-T_{aw}}$ [W/(m${}^{2}$K)]
$A_{CNT}$	Heated CNT surface area [m${}^{2}$]
*d*	Layer thickness [μm]
*D*	Cylinder diameter [mm]
*f*	Frequency [Hz]
γ	Heat capacity ratio [-]
*k*	Thermal conductivity [W/(mK)]
λ	Wavelength [nm]
$Ma_{\infty }$	Inflow Mach number [-]
$p_{\infty }$	Ambient pressure [kPa]
$p_{t}$	Total pressure [kPa]
Pr	Prandtl number [-]
$q_{\infty }$	Dynamic pressure [kPa]
$q_{el}^{\prime \prime }$	Electrically generated heat flux $=U\cdot I/A_{CNT}$ [W/m${}^{2}$]
$q_{cond}^{\prime \prime }$	Conductive heat flux [W/m${}^{2}$]
$q_{conv}^{\prime \prime }$	Convective heat flux [W/m${}^{2}$]
$q_{rad}^{\prime \prime }$	Radiative heat flux [W/m${}^{2}$]
*r*	Recovery factor[-]
$R^{2}$	Determination coefficient of a (linear) fit [-]
$Re_{D}$	Reynolds number based on cylinder diameter *D* = 10 mm [-]
Sr	Non-dimensional frequency; Strouhal number =f·D/u
$t_{i}$	Camera integration time [μs]
$T^{\prime }$	Temperature fluctuation [K]
$T_{aw}$	Adiabatic wall temperature [K]
$T_{t}$	Total temperature [K]
$T_{TSP}$	Surface temperature field measured with TSP [K]
$T_{s}$	Temperature at surface sensor [K]
$T_{w}$	Temperature at wall sensor [K]
*u*	Inflow velocity [m/s]
*x*; x/D	Axial; normalized axial coordinate
*y*; y/D	Span-wise; normalized span-wise coordinate
